# Viability and Diversity of the Microbial Cultures Available in Retail Kombucha Beverages in the USA

**DOI:** 10.3390/foods13111707

**Published:** 2024-05-29

**Authors:** Erin N. O’Sullivan, Daniel J. O’Sullivan

**Affiliations:** Department of Food Science and Nutrition, Microbial and Plant Genomics Institute, University of Minnesota, St. Paul, MN 55108, USA; osull083@umn.edu

**Keywords:** fermentation, probiotic, cultures, amplicon profiling, viable plate count

## Abstract

Kombucha is a two-stage fermented sweetened tea beverage that uses yeast and lactic acid bacteria (LAB) to convert sugars into ethanol and lactate and acetic acid bacteria (AAB) to oxidize ethanol to acetate. Its popularity as a beverage grew from claims of health benefits derived from this vibrant microbial bioconversion. While recent studies have shed light on the diversity of cultures in Kombucha fermentation, there is limited information on the diversity, and especially viability, of cultures in retail beverages that advertise the presence of Kombucha and probiotic cultures. In this study, 12 Kombucha beverages produced by different manufacturers throughout the US were purchased and microbially characterized. Eight of the beverages contained viable Kombucha cultures, while 3 of the remaining 4 had viable *Bacillus* cultures as added probiotics. Amplicon profiling revealed that all contained Kombucha yeast and bacteria cells. The dominant yeasts detected were *Lachancea cidri* (10/12), *Brettanomyces* (9/12), *Malassezia* (6/12), and *Saccharomyces* (5/12). Dominant LAB included *Liquorilactobacillus* and *Oenococcus oeni*, and AAB were *Komagataeibacter*, *Gluconobacter*, and *Acetobacter*. One beverage had a significant amount of *Zymomonas mobilis*, an ethanol-producing bacterium from Agave cactus. While Kombucha beverages differ in the types and viability of cultures, all except one beverage contained detectable viable cells.

## 1. Introduction

Kombucha is a fermented tea beverage composed of a SCOBY (symbiotic relationship of bacteria and yeast), water, tea, and sugar. It is reported to have been originally developed during the Chinese Tsin Dynasty (249–206 BC), where it was thought to be associated with health and longevity [1]. While the precise country of origin is unknown, its current name comes from the Japanese word ‘Kombu’, which refers to *Luminaria japonica*, which was reported to be an edible fungus but is actually a marine alga [2]. It was originally referred to as ‘tea fungus’ due to the surface cellulose-based pellicle that forms during production, resembling a fungal mat [3]. This traditional fermented beverage has also been referred to as Teekvass, Cajnij, Hongo, Teeschwamm, and Wunderpilz in different countries worldwide [4]. Its historical association with nutritional benefits precipitated its subsequent introduction into Japan, Korea, and Eastern Europe, where it gained many legendary health attributes over the centuries [5]. While reports on the global history of Kombucha are not consistent throughout, a common health benefit theme prevails. More recently, it has become popular in many Western countries and has consistently gained popularity in the US market over the last two-plus decades. Its market share in the US reached USD 1 billion in 2021 and is projected to continue growing by 16.2% annually for the next decade [6].

The potential health benefits that give Kombucha its popularity come from the presence of viable cultures of fermentative bacteria and yeast as well as bioactive components such as phenolic compounds and organic acids formed during the fermentation [7]. Like other traditional fermented foods, such as yogurt, kefir, and kimchi, it is viewed as a probiotic food due to the presence of viable cultures [8]. However, Kombucha has not been researched as much as other fermented foods as to if and how the live and active cultures in Kombucha play a role in health benefits. However, the presence of lactic acid bacteria (LAB) has been reported in Kombucha, substantiating its probiotic potential [9,10]. Studies examining the diversity of microbes during Kombucha production have occurred, but few studies have looked at the viability and diversity of cultures in retail Kombucha drinks that are consumed by the public. Recently, six different retail Kombuchas in New Zealand were analyzed, and it was found that only three contained viable cultures [11]. Studies have reported inconsistencies in product labels of retail Kombucha drinks regarding the term ‘probiotic’ and actual viable cultures present, indicating a growing need to further standardize labeling to reflect the probiotic potential of available Kombucha products more accurately [12].

Early microbial studies of Kombucha found that a defined culture of two yeasts and one acetic acid bacterium (AAB) was minimally required for a successful Kombucha fermentation [13]. Typically, there are a lot more fermentative bacteria and yeasts within Kombucha drinks, which likely provide additional potential health benefits and flavors. These include genera of AAB (*Komagataeibacter*, *Gluconobacter*, and *Acetobacter*) [14], LAB (*Lactobacillus*, *Oenococcus*, and *Lactococcus*) [10], and yeasts (*Saccharomyces*, *Zygosaccharomyces*, *Torulaspora*, and *Brettanomyces*) [9]. Defined probiotic cultures are sometimes added in commercial Kombuchas to potentially enhance the nutraceutical aspect of the beverage [12].

The health benefits attributed to Kombucha are impressive, ranging from the prevention of different diseases to enhancing mental attributes [15]. However, human clinical trials are currently very limited and are needed to substantiate the potential health claims from cell culture and animal studies [15,16]. Many studies suggest that Kombucha may offer many holistic benefits regarding colon cancer, diabetes, and inflammatory improvements. For example, an in vitro study by Rasouli et al. (2021) using a cancer cell line HCT-116 compared the use of Kombucha to the anticancer drug doxorubicin for the treatment of colon cancer [17]. They found that Kombucha was able to increase the expression of a variety of genes to overall help slow down and stop the growth of cancer cells more effectively than doxorubicin. Another in vitro study by Xu et al. (2022) used a mouse model to explore the mechanism of how Kombucha could be used to protect against diabetes [18]. They showed a decrease in insulin resistance and improved beta cell function, thus improving diabetes symptoms in mice. Perhaps the most surprising but strongest potential health benefits of Kombucha are its strong anti-inflammatory effects, including lipopolysaccharide (LPS) challenges. Using a mouse model, Wang et al. (2021) showed a strong correlation between Kombucha feeding and reduced tumor necrosis factor-alpha (TNF-α) levels, which is a potent inflammatory cytokine specifically induced by LPS [19]. The study also found a reduction in inflammatory interleukins (IL-1ß and IL-6) and a significant reduction in LPS-induced sepsis in this mouse study. Given that Kombucha fermentation involves high levels of Gram-negative bacteria, which are a source of dietary LPS, this anti-inflammatory effect is very promising. Recent studies examining the ability of dietary Kombucha to modulate the gut microbiota provide a likely mechanism for the observed anti-inflammatory effects that contribute to the potential health benefits [20].

Studies in recent years looking at the diversity of microorganisms in Kombucha fermentations have primarily utilized non-culturing molecular approaches, using either metagenomic or meta-amplicon sequencing. Arikan et al. (2020) used 16S rRNA amplicon sequencing to reveal *Komagataeibacter* as the most prominent bacterial genus from two homemade Kombuchas produced in Turkey [21]. This AAB genus was first described in 2012 and is most closely related to the genus *Gluconacetobacter* [22]. Another study utilized these approaches to examine the microbial diversity in nine retail Kombucha samples purchased in Los Angeles, CA, USA and found *Komagataeibacter*, *Gluconacetobacter*, and *Acetobacter* were the dominant AAB present and *Brettanomyces* was the dominant yeast [23]. While this latter study did not examine culture viability, it was assumed throughout that the cultures detected were viable. Given that inactivated microbial cells can contain DNA for significant but unknown lengths of time, it is important to examine this further. Therefore, the objective of this current study was to combine culturing and non-culturing molecular techniques to examine both the viability and diversity of different Kombucha beverages available in retail stores in the US.

## 2. Materials and Methods

### 2.1. Selection of Kombucha Beverages

Twelve different Kombucha beverages from different manufacturers were purchased in various grocery stores within the Twin Cities (Minneapolis and St. Paul, MN, USA). Table 1 details the 12 Kombuchas and their listed relevant ingredients and processes. The ‘best before’ dates on each beverage were all more than five months from the date of purchase. These represent all the major brands available in this market, as well as the brands with the largest market share in the USA [23]. All samples were maintained at 4 °C and used for culturing studies within two weeks of purchase.

### 2.2. Viable Culture Analysis

The following selective and non-selective media were used for quantitative culture analysis of the kombucha samples. BHI (Brain Heart Infusion) agar media was used as a general non-selective medium. MRS (de Man–Rogosa–Sharpe) medium was used for lactic acid bacteria (LAB) and *Bacillus* probiotics. Acetic acid bacteria (AAB) selective medium (ABS) [24] was used for growth of AAB. Potato Dextrose Agar (PDA) supplemented with 25 mg/mL chloramphenicol was used for selective growth of yeast (PDA-Y). MacConkey agar (MAC) plates were used to test for the presence of any fecal coliform bacteria. Serial 1:10 dilutions of each Kombucha drink were generated using sterile peptone water up to 10^−4^ and spread-plated onto each agar medium. Both dilutions and platings were conducted in duplicate to ensure the reproducibility of the values reported. All plates were incubated at 30 °C aerobically for both bacteria and yeast, and MAC plates were also incubated at 37 °C. After 48 h, colony-forming units (CFU) were calculated and representatives of different types of microbes were inoculated into BHI broth media for further analysis.

### 2.3. Analytical Techniques

Gram stains were conducted using freshly grown cultures for each of the different microorganisms representing different colony types from different agar media and viewed under a light microscope at 1000× magnification. All pH measurements of fresh Kombucha beverages were obtained with newly calibrated pH probes using a Mettler Toledo SevenEasy instrument (Columbus, OH, USA). Measurements were performed in duplicate to confirm reproducibility within the values reported.

### 2.4. Molecular Identification of Select Kombucha Colony Isolates

Colonies were inoculated into BHI broths, and when fully grown, 1.5 mL of culture was pelleted using an Eppendorf centrifuge and the supernatant was completely removed. Pellets were then resuspended in 500 µL molecular grade water and 200 µL glass beads (<106 µm diameter; Sigma, St Louis, MO, USA), and cells were disrupted in a Minibeadbeater-8 (Biospec Products, Bartlesville, OK, USA) for 20 s at maximum speed. Tubes were immediately placed on ice for debris to settle, and 100 µL of the DNA-enriched supernatant was transferred to a fresh tube and diluted 1:10 to provide the template for PCR.

The 16S rRNA gene was amplified using primers: 27F, 5′-AGAGTTTGATCMTGGCTCAG-3′ and 1525R, 5′-AAGGAGGTGATCCAGCC-3′ with *Taq* DNA polymerase in a reaction volume of 50 µL using a Robocycler (Stratagene, La Jolla, CA, USA) with the following conditions: 1 cycle of 92 °C for 2 min and 35 cycles of 92 °C × 1 min; 50 °C × 30 s and 72 °C × 1 min. One percent agarose gels in 1× TAE buffer (Bio-Rad, Hercules, CA, USA) were used for electrophoresis of PCR samples. Bands were cut out, and the DNA was gel extracted using a kit from New England BioLabs (Ipswich, MA, USA) according to the manufacturer’s instructions. Purified DNAs were quantified using a Nanodrop spectrophotometer (Thermo Scientific, Waltham, MA, USA), and Sanger sequencing of amplicons was conducted at ACGT DNA Sequencing Services (Wheeling, IL, USA).

### 2.5. Amplicon Profiling of Kombucha Beverages

Fresh beverages were initially mixed by shaking to ensure microbial cells were properly suspended. Five mL of samples were pelleted by centrifugation to pellet any microbial cells. Pellets were resuspended in 1 mL molecular grade water and pelleted by centrifugation to ensure removal of all liquid. Pellets were then resuspended in warm MBL buffer from the DNeasy PowerFood Microbial DNA Isolation Kit (Qiagen, Redwood City, CA, USA). The manufacturer’s instructions were followed for total DNA isolation, except the disruption of cells using the PowerBead tubes was conducted with a Minibeadbeater-8 (Biospec Products, Bartlesville, OK, USA) for 20 s at maximum speed. The V3–V4 region of the bacterial 16S rRNA gene and the ITS1 (region between the fungal 18S and 5.8S rRNA genes) were used for profiling the total bacterial and yeast diversity in each Kombucha. All amplifications and paired-end sequencing reactions were conducted at the University of Minnesota Genomics Center using techniques previously described [25].

Four FASTQ raw sequence files were generated for each sample corresponding to the paired-end reads for each amplicon. Trimming and merging of paired sequences was conducted using Geneious Prime^®^ 2024.0.3 Software [26]. Sequences were further quality controlled using the BBDuk plugin from the Joint Genome Institute (Department of Energy) in this software using a minimum sequence quality score of 32. Following the assembly of identical sequences into taxonomical groups, species identifications were assigned to each group using BLAST database searches from the NCBI (National Center for Biotechnology Information). Relative abundances were assigned based on the number of sequence reads corresponding to each taxonomic grouping.

## 3. Results

### 3.1. Viable Culture Analysis of Kom 1–Kom 12

Fermented foods have a long tradition of containing potentially healthy bioactive compounds produced from fermentation as well as live microbial fermentative cultures. While fermented dairy beverages have a long history of containing live and active fermentative cultures, there is considerably less known about the viability of cultures available in commercially available Kombucha beverages. To investigate this, 12 Kombucha beverages were examined for relevant Kombucha cultures, including yeast, AAB, LAB, and added probiotics (where relevant). Table 2 details the viable plate counts of the 12 Kombuchas on various media as well as the pH measurements of each. Kom 11 was the only product that contained no detectable viable cells on any plate. All other products contained viable cells at levels of 10^3^ to 10^7^ cfu/mL. However, 3 of these Kombucha’s (Kom 1, Kom 3, and Kom 6) did not have any viable Kombucha fermentative cultures (AAB, LAB, or yeast) but did contain added *Bacillus* cultures as probiotics. Two of the products (Kom 2 and Kom 7) contained colonies on MacConkey agar plates, which were included as they are routinely utilized to test for fecal coliform bacteria in the food industry.

### 3.2. Microscopic Analysis of Different Cultures Isolated from the Selective Media

**Figure 1:**
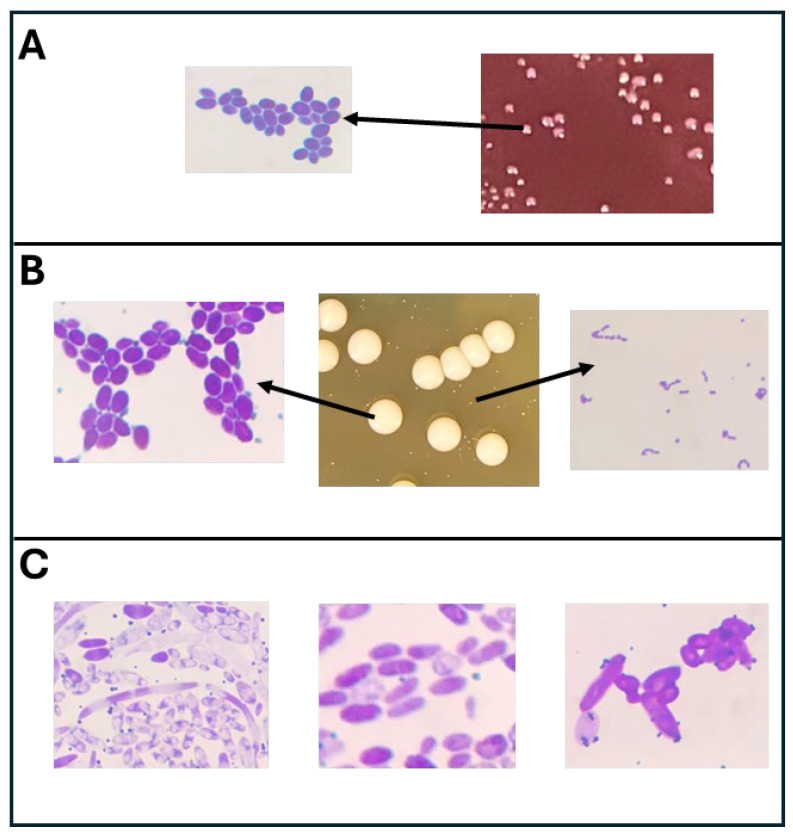
(**A**): Isolated colonies from a MAC plate of a 10^0^ dilution of Kom 7 with arrow pointing to microscopic view of the indicated colony. (**B**): Isolated colonies from an MRS plate of a 10^−3^ dilution of Kom 12 with arrows pointing to microscopic views of the indicated colonies. (**C**): Microscopic views of colonies depicting different yeast cellular morphologies from various Kombuchas.

Selected colonies from different Kombuchas and different agar media were cultured in BHI broths for Gram staining and microscopic analysis. This revealed that at least some yeast could grow on all agar media, including MacConkey agar. The two Kombucha samples that had growth on this medium (Kom 2 and Kom 7) both exhibited similar colony morphologies, suggesting a single type of yeast species. Microscopic analysis of these colonies also revealed similar morphologies substantiating this result (Figure 1A). In addition, the MRS medium, which is selective for LAB, also showed yeast growth as well as viable Gram-positive coccoid cells in chains, indicative of LAB (Figure 1B). This distinctive budding yeast morphology was also seen in different Kombucha’s as well as from colonies on the AAB selective medium, ACS. A short Gram-negative rod that came from a colony on the ACS plate from Kom 4 was used for PCR amplification of its 16S rRNA gene, and sequence analysis of this gene indicated it was an AAB, *Acetobacter tropicalis*, which is consistent with its observed morphology. While most yeast morphologies observed microscopically were single cells, some elongated/filamentous cellular structures were also seen (Figure 1C).

### 3.3. Bacterial and Fungal Diversity Obtained from Direct Amplicon Profiling of Each Kombucha

**Figure 2:**
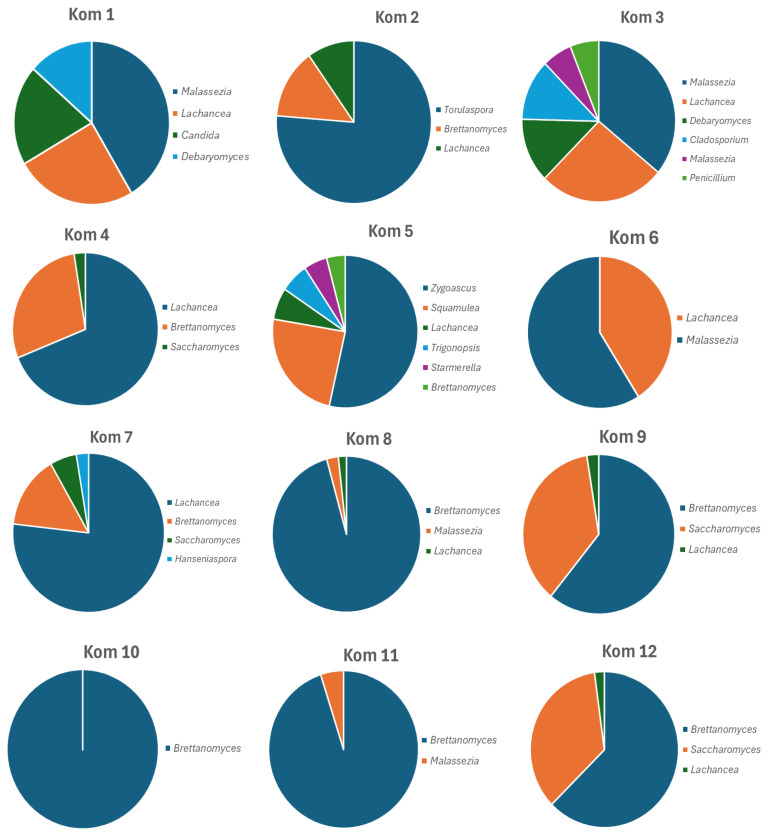
Graphical representation of the numerically dominant genera of yeast cells identified in the 12 Kombucha beverages from ITS amplicon profiling.

Kombucha production involves both anaerobic and aerobic fermentation activities, whereby sugars are first fermented to ethanol, primarily by yeast, and to organic acids by LAB. Subsequently, the AAB aerobically converts ethanol to acetic acid, and, therefore, the functional Kombucha cultures are composed of both fungal (yeast) and bacterial components (LAB and AAB). While 4 of the 12 Kombucha’s did not have any detectable viable bacteria or yeast fermentative cultures, the amplicon profiling did reveal Kombucha cultures for all 12 of the samples, indicating that intact non-viable cells were present. The relative abundance of different amplicon sequences gave a good picture of the dominant types involved, and some samples were much more diversely represented than others. It should be noted that diversity in a specific food fermentation is significantly lower than microbial diversity in a dynamic natural ecosystem, such as soil or feces, and often may be dominated by one or more genera. Figure 2 compares the dominant fungal genera observed in the 12 Kombucha samples and illustrates this differential; many samples are primarily dominated by one or two genera, while others are less so. The *Brettanomyces* yeast genus was the most dominant in 5 samples (Kom 8–Kom 12) and was also a significant member of 4 of the other 7 samples. Two species were represented, *B. bruxellensis* the most common, and *B. anomalus* (Table 3). The other most prominently represented dominant yeast genus was *Lachancea*, which was found in 10 of the 12 samples and represented the most dominant yeast genus in Kom 4 and Kom 7 (Figure 2). It was represented by only one species, *L. cidri*, indicating the prevalence of this yeast species in Kombucha brewing in the USA currently. The prominent ethanol-producing yeast genus *Saccharomyces* was present in 5 samples, with *S. cerevisiae* the most common species, and was one of the two most dominant yeast species in Kom 9 and Kom 12. The overall relative abundance in the 12 Kombuchas, using a minimum abundance of 1% per sample, revealed 24 different yeast/fungal species, representing 18 genera (Table 3).

**Figure 3:**
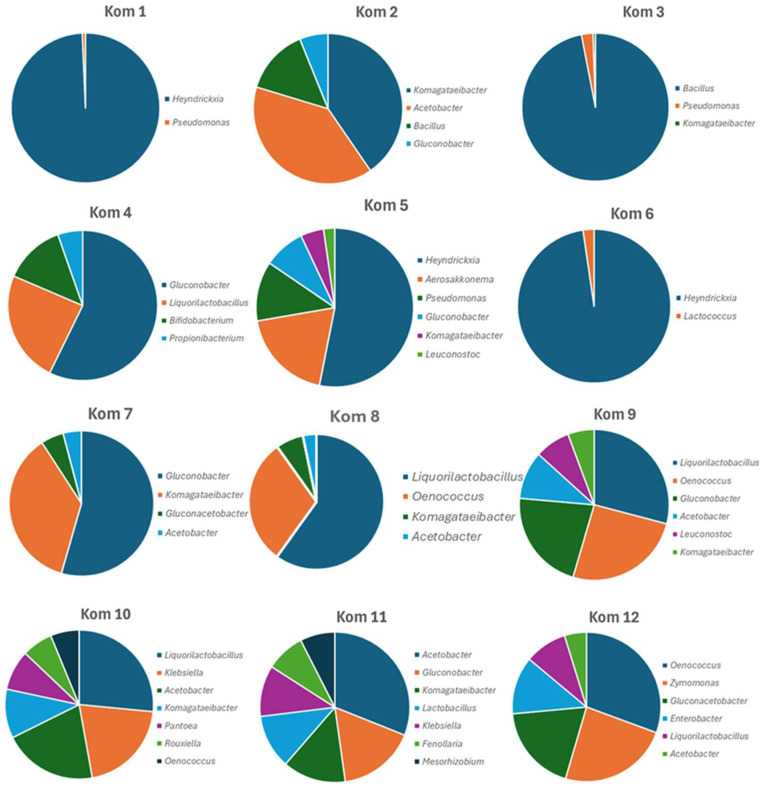
Graphical representation of the numerically dominant genera of bacterial cells identified in the 12 Kombucha beverages from 16S rRNA gene amplicon profiling.

A total of 5 of the 12 Kombucha beverages listed added ‘*Bacillus*’ probiotics as an ingredient. Kom 1, Kom 5, and Kom 6 listed *B. coagulans*, while Kom 2 and Kom 3 listed *B. subtilis*. In a recent reclassification of some *Bacillus* species and species of other genera, *B. coagulans* was included in the new genus *Heyndrickxia* and thus is represented in the BLAST database under this name [27]. The 16S rRNA amplicon profiling was consistent with this as the added culture was dominant in all cases (Figure 3). In Kom 1, Kom 3, and Kom 6, it was so numerically dominant that the AAB and LAB were essentially not represented in Table 4 for these samples as it was limited to 1% relative abundance. However, the expected repertoire of AAB and LAB were present at <1% relative abundance in these samples.

The major AAB genera represented were *Acetobacter*, *Gluconobacter*, *Gluconacetobacter*, and *Komagataeibacter*, which is consistent with other studies on Kombucha. The most dominant AAB genera in each Kombucha sample were *Gluconobacter* (Kom 1, Kom 4, Kom 5, Kom 6, Kom 7, and Kom 9), *Komagataeibacter* (Kom 2, Kom 3, and Kom 8), *Acetobacter* (Kom 10 and Kom 11), and *Gluconacetobacter* (Kom 12). Of the detected LAB genera, *Liqorilactobacillus* and *Oenococcus* were the two genera with significant relative abundance in many of the Kombuchas (Kom 4, Kom 8, Kom 9, Kom 10, and Kom 12) (Figure 3). *Lactobacillus*, *Leuconostoc*, and *Lactococcus*) were represented at lower abundances in a few others (Kom 5, Kom 6, and Kom 11). The overall diversity of bacteria in the 12 Kombuchas was greater than for fungi, with 45 species representing 34 genera (Table 4).

## 4. Discussion

Kombucha has become an increasingly popular beverage at retail outlets in the US, primarily due to its perceived health benefits from the bioactive components produced by the yeast and bacterial cultures during its fermentation process. This process is a two-stage fermentation process whereby yeast and LAB first ferment simple sugars into ethanol and lactic acid, and subsequently, AAB aerobically oxidizes ethanol into acetic acid. The final beverage should be acidic and protected from foodborne bacterial pathogens. Two previous studies on retail available Kombucha reported pH ranges of beverages in New Zealand as 3.2–3.9 [11] and in Los Angeles, CA as 3.0–3.2 [23]. In this current study, the pH ranges of the 12 Kombucha beverages analyzed were 3.0–3.8, illustrating the protective acidic profile of these beverages against food pathogens. The appearance of colonies on MacConkey agar plates for Kom 2 and Kom 7 was initially surprising as this medium and variants thereof are widely used in food microbiology laboratories for the detection of fecal coliform *Enterobacteriaceae* [28,29]. However, some lactose fermenting strains of yeast with tolerance to bile salts can also form colonies on this medium [30].

The presence of viable cultures in Kombucha beverages depends on their acid tolerance over time. While fermentative yeasts exhibit a higher tolerance than fermentative bacteria in general, many AAB and LAB can withstand acid environments for different time periods, with some AAB having evolved higher tolerances to acetic acid [31]. Yeasts and AAB are frequently isolated from Kombucha, including commercial beverages, demonstrating their survival abilities under these conditions [11]. This is consistent with findings in this study based on microscopic analysis of colonies obtained on different media. The molecular identification of one of these isolates from Kom 4, which exhibited a Gram-negative rod phenotype, indicated it was a strain of *Acetobacter tropicalis*. While this species was not detected in the major AAB of Kom 4 using the direct amplicon profiling (Table 4), it was present at lower relative abundances (0.2% of total reads), demonstrating the differential tolerance of individual AAB to acetic acid. LAB are generally more sensitive to prolonged exposure to pH < 4.0, and LAB-fermented vegetable foods that are allowed to ferment until completion, such as sauerkraut, rapidly lose the viability of LAB. However, some LAB can remain viable in the pH range 3–4 for extended periods. While most studies that examined viable cultures in Kombucha focused on AAB and yeast, some LAB genera such as *Pediococcus* have been isolated from Kombucha [32]. In this current study, a colony from an MRS plate of Kom 12 exhibited Gram-positive cocci cells arranged in chains, which is indicative of some LAB genera (Figure 1B). However, as *Oenococcus oeni* was the most dominant bacterial culture identified from the amplicon profiling of Kom 12 (Figure 3), it is most likely this organism. This is best known as an acid-tolerant LAB organism important for the malolactic conversion of malic acid to lactic acid during the aging phase of wine production [33]. It has also been frequently isolated from Kombucha [34,35].

The dominant fungal genus in most Kombucha beverages identified in this study was *Brettanomyces*, which was detected in 9 of the 12 samples above the 1% relative abundance level (Table 3). This is consistent with microbial studies on Kombucha since first reported in a study by Mayser et al. (1995), where it was identified in 56% of samples tested [2]. Since then, it has been one of the more frequent genera reported from Kombucha studies throughout the world. Another dominant yeast identified among the Kombucha beverages in this study was *Lachancea cidri*, which was detected in 10 of the 12 samples at significant levels (Figure 2). This species has not been reported previously as part of a Kombucha microbial consortium and is more frequently found in cider fermentations [9,36]. However, another species of *Lachancea*, *L. fermentati*, has been found in a few Kombucha studies [37,38,39]. The fungus *Malassezia* with yeast-like cells was also a notable component in 6 of the 12 Kombucha beverages and represented >35% of the relative abundance of Kom 1, Kom 3, and Kom 6. This fungus has also been reported as a component of Kombucha microbial studies previously [40,41]. The natural habitat for *Malassezia* fungi is the skin of humans and animals, where it is frequently associated with various dermatological disorders [42]. Its presence in some Kombucha beverages at such high levels is surprising. *Torulaspora microellipsoides*, an ethanol-producing yeast of the *Saccharomycetaceae* family, was the major fungal representative of only one sample, Kom 2 (66.5% abundance level). While not frequently reported as a dominant yeast component for Kombucha, it was detected at low levels in Kom 3 as well as in previous Kombucha studies [9]. Given that it is an environmental yeast often associated with viticulture, its dominance in a Kombucha fermentation is not surprising and illustrates the diversity of potential functional yeast varieties that can form the basis of a Kombucha culture.

Yeasts are the primary ethanol producers in all Kombucha fermentations and were detected in all Kombucha beverages in this study. However, Kom 12 was unique in that it also had a significant proportion of its bacterial population (22.6% relative abundance) composed of the ethanol-producing Gram-negative bacterium *Zymomonas mobilis* [43]. This bacterium is found on certain plants, such as the Agave cactus in Mexico, and is best known for its involvement in the primary ethanol fermentation of Agave during mezcal, pulque, and tequila production [44,45,46]. Its presence in the SCOBY used for the Kom 12 fermentation may reflect insights into its origin. *Liquorilactobacillus* was the most numerically dominant bacterial genus detected in Kom 8, Kom 9, and Kom 10 (Figure 3). This LAB has previously been seen to predominate in a Kombucha microbial consortia during its evolution over 3 years and may reflect beverages produced with a mature SCOBY inoculum rather than a freshly generated one [47]. This is also reflective of its relatively high tolerance to acid compared to other LAB and is also of interest for its potential probiotic characteristics [48].

**Figure 4:**
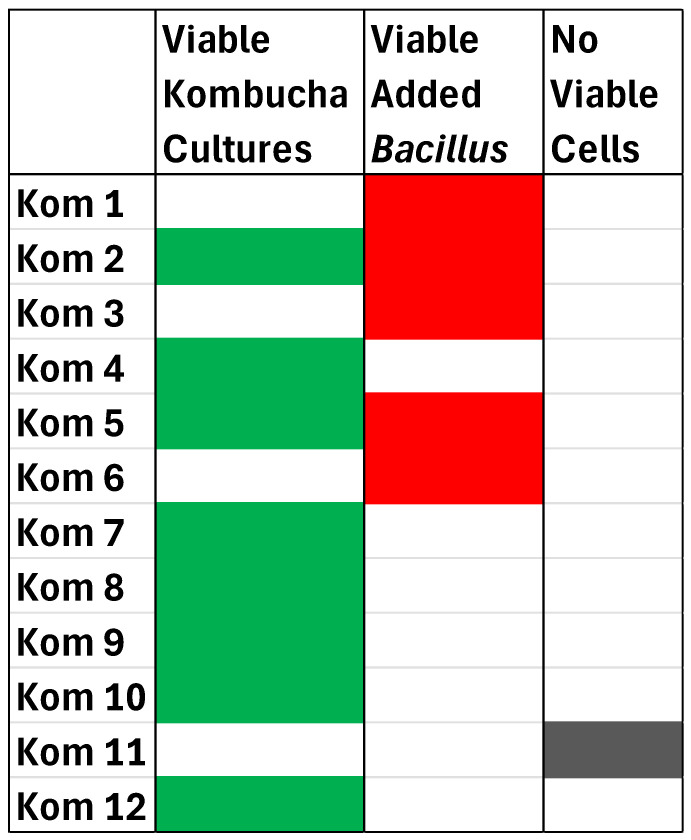
Viable culture profile of the 12 Kombucha beverages analyzed in this study. Green represents viable Kombucha cultures (Yeast, AAB, and LAB). Red represents viable added *B. coagulans* or *B. subtilis*. Gray indicates no viable cells detected.

The term ‘probiotic’ was used on the beverage container in 10 of the 12 Kombuchas (Table 1). This term implies the ingestion of live microorganisms to confer a health benefit on the host and was first used in this context by Fuller (1989) [49]. While the use of the term by different authors has had numerous variations, all comply with ingesting a viable organism that can positively modulate the intestinal microflora of the host to confer a health benefit [50]. While four of the Kombuchas did not have viable Kombucha fermentative cultures, likely due to pasteurization or other processing steps to terminate the fermentation, three of them did contain viable *Bacillus* cultures as probiotics (Figure 4). As these are endospore formers, they would survive these processing steps. It is ironic that the one beverage that did not contain any detectable viable organisms (Kom 11) used the brand name ‘Live Probiotic Kombucha’. This highlights the need for more regulatory oversight of the accuracy of food labels pertaining to probiotics in the US.

## Figures and Tables

**Table 1:** Commercial Kombucha beverages used in this study.

Kombucha Beverage	State	Relevant Ingredients Listed Regarding Microbial Fermentation	Relevant Label Claims
Kom 1 ^b^	CA	Kombucha cultures, *Bacillus coagulans* MTCC5856, cane sugar, black tea, green tea, lime juice, and grape juice	“Bubbly probiotic tea”
Kom 2 ^b^	WA	Kombucha culture, *Bacillus subtilis*, cane sugar, tea blend, ginger juice, whole lemon puree	“2 billion CFU Probiotic Cultures”
Kom 3 ^b^	OR	Kombucha cultures, *Bacillus subtilis*, green tea, black tea, cane sugar, apple, white grape and passion fruit juice, mango puree	“Probiotic Kombucha” “2 billion Probiotic Cultures”
Kom 4 ^b^	OR	Kombucha culture, currant, oolong tea, elderberry, hibiscus, blueberry, goji berry, strawberry, raspberry, cane sugar	“Fizzy Probiotic Tea”
Kom 5 ^b^	CA	Kombucha culture, black tea, green tea, kiwi and ginger juice, *Bacillus coagulans* GBI-306086	“9 billion Living Probiotics”
Kom 6 ^b^	CA	Kombucha culture, *Bacillus coagulans* MTCC 5856, black and green tea, cane sugar, stevia leaf extract, green coffee bean extract	“Live Probiotics”
Kom 7 ^b^	IL	Black tea, cane sugar, and cherry and lemon juice concentrate	“Live Probiotics & Enzymes” “Heirloom cultures”
Kom 8 ^c^	MN	Black and green tea, cane sugar, apple juice	“Live Probiotics” “Active Kombucha Culture”
Kom 9 ^c^	WI	Kombucha culture, green tea, hibiscus flowers, cane sugar	“Forage Kombucha is Alive” “Kombucha Culture”
Kom 10 ^b^	MN	Peach oolong tea, mango turmeric tea, cane sugar	“Non-pasteurized” “Fresh Brewed”
Kom 11 ^b^	TX	Kombucha culture, sugar, stevia extract	“Live Sparkling Probiotic Kombucha”
Kom 12 ^b^	WI	Kombucha culture, black tea, safflower, cane sugar	“Contains millions of living probiotic cultures” “Live Kombucha Culture”

^b^, bottled beverage; ^c^, canned beverage.

**Table 2:** pH readings and viable microbial plate counts obtained on the different agar media for the 12 Kombucha beverages.

Kombucha Sample	pH Reading	Total CFU BHI (cfu/mL)	Total CFU MRS (cfu/mL)	Total CFU ABS (cfu/mL)	Total CFU PDA-Y (cfu/mL)	Total CFU MAC (cfu/mL)
Kom 1	3.2	3.2 × 10^5^	3.5 × 10^5^	nc ^1^	nc	nc
Kom 2	3.5	3.4 × 10^6^	3.1 × 10^6^	1.7 × 10^5^	1.7 × 10^5^	1.0 × 10^4^
Kom 3	3.7	5.0 × 10^5^	2.5 × 10^6^	nc	nc	nc
Kom 4	3.3	3.0 × 10^3^	5.5 × 10^3^	1.2 × 10^4^	1.2 × 10^4^	nc
Kom 5	3.1	1.1 × 10^4^	3.3 × 10^3^	7.0 × 10^3^	1.1 × 10^4^	nc
Kom 6	3.2	1.0 × 10^3^	1.0 × 10^3^	nc	nc	nc
Kom 7	3.3	5.5 × 10^5^	9.7 × 10^5^	8.5 × 10^5^	4.7 × 10^5^	3.0 × 10^3^
Kom 8	3.6	2.0 × 10^5^	6.3 × 10^5^	5.2 × 10^6^	1.5 × 10^6^	nc
Kom 9	3.0	2.4 × 10^6^	1.5 × 10^6^	1.6 × 10^6^	2.0 × 10^6^	nc
Kom 10	3.4	1.1 × 10^6^	2.2 × 10^6^	3.8 × 10^6^	6.0 × 10^6^	nc
Kom 11	3.5	nc	nc	nc	nc	nc
Kom 12	3.8	2.3 × 10^6^	1.9 × 10^6^	3.1 × 10^6^	2.1 × 10^6^	nc

^1^, no colonies.

**Table 3:** Relative abundance of fungal species identified (minimum 1%) from amplicon profiling for each Kombucha sample.

Fungal Species	Kom 1	Kom 2	Kom 3	Kom 4	Kom 5	Kom 6	Kom 7	Kom 8	Kom 9	Kom 10	Kom 11	Kom 12
*Allodekkera sacchari*		7.1										
*Brettanomyces anomalus*							14.7	4.1	11.8	3.5		35.2
*B. bruxellensis*		12.2		26.5	3.8			88.7	48.9	95.1	94.5	26.6
*Candida mesenterica*	5			1.6								
*C. parapsilosis*	14											
*Cladosporium angustiherbarum*	3		11.5									
*Cyberlindnera jadinii*		3.5										
*Debaryomyces psychrosporus*	13		12.2									
*Gibellulopsis serrae*					1.2							
*Hanseniaspora valbyensis*							2.6					
*Lachancea cidri*	24.1	8.5	25	67.8	6.2	38.5	75.5	1.7	2.5			2.1
*Malassezia arunalokei*	14.5				1.6	38.5						
*M. globosa*			5.9			14.4		2.5				
*M. restricta*	25.2		33.8		2.4	2.4					2	
*M. slooffiae*											3	
*Penicillium hordei*			5.7									
*Pichia cecembensis*			1.8									
*Saccharomyces bayanus*				2.5								
*S. cerevisiae*			2.1				5.6		36.5			34.8
*Squamulea flakusii*					22.2							
*Starmerella davenportii*					4.8							
*Torulaspora microellipsoides*		66.5	2									
*Trigonopsis variabilis*					5.8							
*Zygoascus hellenicus*					49.5							

**Table 4:** Relative abundance of bacterial species identified (minimum 1%) from amplicon profiling for each Kombucha sample.

Bacterial Species	Kom 1	Kom 2	Kom 3	Kom 4	Kom 5	Kom 6	Kom 7	Kom 8	Kom 9	Kom 10	Kom 11	Kom 12
*Acetobacter ascendens*								3	9.5			
*A. musti*		34.1										
*A. suratthaniensis*							1.5					
*A. tropicalis*							2.4			19	19.2	4.5
*Acidomonas methanolica*					1							
*Aerosakkonema funiforme*		3			16.7							
*Bacillus subtilis*		12.2	94.8									
*Bifidobacterium minimum*				11.9								
*Bosea vaviloviae*											2	
*Brochothrix thermosphacta*					1.6						4.1	
*Clostridium saudiense*											1.4	
*Enterobacter cloacae*												11.8
*Escherichia fergusonii*											1.3	
*Fenollaria massiliensis*											5.2	
*Gluconacetobacter diazotrophicus*							4.8					
*G. entanii*												17.9
*G. takamatsuzukensis*		4										
*Gluconobacter japonicus*											10.4	
*G. oxydans*		5.3		51.5	7.4		51.7		20.2			
*Heyndrickxia coagulans*	97.9				46.4	94.2	1.2				2.2	
*Hoylesella timonensis*											1.3	
*Klebsiella variicola*										19.2	6.8	
*Komagataeibacter rhaeticus*					4.1		34.5	6.2	5.3	9.9		
*K. saccharivorans*		35.1									8.4	
*Kosakonia radicincitans*												1
*Lacticaseibacillus brantae*								1.2				
*Lactobacillus delbrueckii*											7.2	
*Lactococcus cremoris*					1.2	2.2					1.6	
*Leuconostoc mesenteroides*									7.1			
*L. pseudomesenteroides*					2							
*Ligilactobacillus faecis*				1.8								
*Liquorilactobacillus aquaticus*								33.5				
*L. hordei*								22.8				8.6
*L. nagelii*				21.7					26.9	24.6	3	
*Mesorhizobium norvegicum*											4.6	
*Mixta gaviniae*										1.8		
*Oenococcus oeni*								28.3	23.6	5.8	2	28.8
*Pantoea brenneri*										8.1		
*Propionibacterium cyclohexanicum*				4.8								
*Pseudomonas weihenstephanensis*			2.5		10.6	1					3.1	
*Rouxiella badensis*											1.3	
*R. chamberiensis*										6.2		
*Sporolactobacillus pectinivorans*				1.5								
*Streptococcus thermophilus*											3	
*Zymomonas mobilis*												22.6

## Data Availability

The original contributions presented in the study are included in the article, further inquiries can be directed to the corresponding author.
